# Renal Resistive Index Along the Renal Arterial Tree: Do Kidney and Cardiovascular Associations Depend on the Measurement Site? A Scoping Review

**DOI:** 10.3390/jcm15218466

**Published:** 2026-10-30

**Authors:** Julia Bryłowska, Tymoteusz Lubas, Arkadiusz Lubas

**Affiliations:** 1 Faculty of Medicine, University of Warsaw, 02-089 Warsaw, Poland; 2 Faculty of Medicine, Medical University of Gdańsk, 80-210 Gdańsk, Poland; 3 Department of Internal Diseases, Nephrology and Dialysis, Military Institute of Medicine—National Research Institute, 04-141 Warsaw, Poland

**Keywords:** renal resistive index, cardiovascular factors, kidney function, renal arterial tree, Doppler ultrasonography, hemodynamics

## Abstract

**Objective:** The renal resistive index (RRI), measured by Doppler ultrasonography, is commonly used to assess renal vascular-interstitial damage. However, the RRI may be associated with cardiovascular and general hemodynamic factors, as well as with structural and functional changes within the kidney. These associations may be influenced by the measurement location of the RRI. This scoping review aimed to evaluate differences in RRI associations with hemodynamic or structural and functional alterations in the kidney across measurements at different sites along the renal arterial tree. **Methods:** A search of the PubMed database was conducted to identify studies evaluating the association between the RRI measured at different levels of the renal arterial tree. Separate search strategies were used for the main renal artery, segmental/interlobar arteries, and intraparenchymal cortical vessels. Studies were selected based on predefined inclusion and exclusion criteria. **Results:** A total of 888 publications were identified. After applying the exclusion criteria, the final analysis included 6 papers on main arteries, 30 on segmental arteries, and 7 on parenchymal arteries. All analyzed publications showed associations between the RRI and cardiovascular or renal factors, or both. **Conclusions:** Available data suggest that the relationship between the renal resistive index and systemic or local changes may vary by measurement site. However, reliable studies directly comparing RRI associations across measurement sites are lacking. Therefore, current evidence does not establish whether RRI measurements obtained at different sites can be used interchangeably.

## 1. Introduction

The renal resistive index (RRI) is calculated from Doppler ultrasound measurements as the ratio of the difference between peak systolic and end-diastolic blood flow velocities to the peak systolic velocity, obtained from the Doppler spectrum of intrarenal arteries, such as the segmental or interlobar arteries [1]. In adults, normal RRI values typically range from 0.47 to 0.70, and the difference between the indices measured in the two kidneys usually does not exceed about 5–8% [2]. The RRI is widely used as a non-invasive marker of renal hemodynamics and has gained increasing clinical importance in recent years. Importantly, the RRI should not be interpreted as a direct measure of renal vascular resistance alone, as a study conducted by Bude et al. [3] demonstrated that it is also influenced by vascular compliance and its interaction with vascular resistance. The RRI has predictive importance in chronic kidney disease, particularly in determining the effectiveness of renal artery stenosis revascularization and the development of the graft in renal transplantation. It has been suggested that low (<0.6) RRI values may be associated with renal artery stenosis (RAS), whereas higher (>0.7) values may reflect vasoconstriction and arteriolosclerosis [3]. Furthermore, in some conditions, the RRI can be crucial for predicting acute renal damage in individuals who are critically ill [4]. Moreover, the RRI is linked to subclinical indicators of target organ damage in essential hypertension and indicates the advancement of renal illness beyond albuminuria and creatinine clearance [5]. As demonstrated in the study by Bigé et al., an RRI ≥ 0.65 was associated with severe interstitial fibrosis, arteriosclerosis, and decline in renal function [6]. Therefore, the RRI may provide information not only about renal microcirculation but also about broader cardiovascular alterations.

Furthermore, the renal resistive index reflects not only intrarenal vascular resistance or stiffness of renal arteries, but also a broader spectrum of pathological and hemodynamic processes, including structural changes within the renal parenchyma and systemic vascular alterations [7]. An example of such systemic vascular involvement was demonstrated in a study by Ohta et al., who evaluated 185 patients and compared resistive index values in renal and carotid arteries [7]. The authors observed that the resistive indices in these two vascular beds increased in parallel to some extent, suggesting that resistive indices may reflect generalized vascular changes rather than organ-specific alterations alone. However, the risk factors associated with increased resistive index differed partially between the renal and carotid arteries, indicating that local and systemic mechanisms may contribute differently to vascular damage. Moreover, under stable conditions, the renal resistive index appears predominantly influenced by factors such as age, pulse pressure, and arterial stiffness, whose progression may further increase in the presence of concomitant diseases, including diabetes or chronic kidney disease [2].

However, the available studies suggest that the clinical significance of the renal resistive index may vary depending on the measurement site within the renal arterial tree. Some reports indicate stronger associations with renal parenchymal damage and biopsy findings when the RRI is measured in segmental/interlobar renal arteries [7]. In contrast, measurements obtained in more proximal, main renal vessels may be more strongly influenced by systemic hemodynamic parameters [8]. This relationship was demonstrated in a study conducted by Prencipe et al. in which the renal resistive index measured in the main renal arteries was compared with echocardiographic parameters of cardiac diastolic function. A significant correlation was found between RRI and transmitral flow deceleration time, with higher RRI values observed in patients with prolonged deceleration time (>240 ms). The RRI threshold that best discriminated patients with impaired diastolic function was approximately 0.675, and progressive increases in deceleration time were associated with corresponding increases in the RRI. These findings support the concept that the RRI assessed in proximal renal vessels may reflect central hemodynamic alterations and cardiovascular function rather than intrarenal resistance alone [9]. Regarding arteries within the renal cortex, Martinoli et al. in their comparative study showed that the RRI values did not differ significantly at the interlobular level [10]. Based on these observations, we hypothesized that the distance of the measurement site from the renal parenchyma may influence the physiological interpretation of the RRI. Specifically, measurements obtained closer to the renal parenchyma may better reflect renal functional impairment and histopathological changes, whereas measurements performed in more proximal arteries may show stronger associations with central hemodynamic parameters such as arterial pressure, pulse pressure, or arterial stiffness. Furthermore, this hypothesis could be supported by the fact that atherosclerosis and macroangiopathy predominantly affect large and medium-sized arteries in response to systemic pressure overload. In contrast, smaller intraparenchymal vessels are less affected by typical atherosclerotic changes [11]. Moreover, the study by Mompeó-Corredera et al. revealed that the renal artery has a different structure depending on the part examined [12]. Its wall composition changes from an elastic vessel to a muscular artery as it goes toward the renal hilum. The tunica adventitia is composed of connective tissue and elastic fibers, but longitudinal smooth muscle cells are present only in the middle and distal parts. The tunica intima shows greater expansion in the proximal areas. Furthermore, according to this study, the tunica media has an equal percentage of muscle fibers along its entire length, and only the proportions of the extracellular matrix, consisting of elastic laminae, change, with most of them in the proximal part of the artery. Also, atherosclerosis is more commonly found in elastic arteries than in muscular ones, making it more likely to develop in the proximal segment of the renal artery. Conversely, Sato et al. evaluated the severity of renal arterial sclerosis and renal glomerular hyalinization in diabetic patients and found that the more distal segments of the renal arterial tree in diabetic nephropathy showed greater severity of arteriosclerosis [13]. Moreover, in the interlobar, arcuate, and interlobular arteries, a positive correlation was observed between glomerular changes and arteriosclerosis. This may suggest diverse involvement of individual parts of the renal artery in diabetes, with a greater impact on its distal parts and smaller branches.

The RRI, which can be easily calculated during Doppler ultrasonography, is widely used in clinical practice and has been extensively described in the literature as a potential marker of renal hemodynamics and renal damage. However, previous studies and reviews, including the review by Darabont et al., have reported associations between the RRI and renal and cardiovascular factors, without specifically examining whether these associations vary by the RRI measurement site [4]. Therefore, further evaluation of the RRI may provide additional insights into its potential role in the non-invasive assessment of renal function and injury. In particular, the selection of the measurement site within the renal arterial tree may influence the diagnostic and prognostic value of this parameter. A better understanding of these relationships could improve the clinical utility of the RRI and enable the identification of preliminary prognostic indicators of renal damage before renal biopsy results are available. Moreover, comparing RRI values obtained from different vascular segments may provide valuable information about their organ specificity and could serve as a basis for future prospective studies investigating the clinical significance of measurement location in the assessment of renal disease.

## 2. Materials and Methods

Between 27 March 2026 and 2 June 2026, a search of the PubMed database was performed to identify studies evaluating the renal resistive index measured at different levels of the renal arterial tree. Separate search strategies were applied for the main renal artery, segmental/interlobar arteries, and intraparenchymal cortical vessels. No review protocol was registered for this scoping review. Data were extracted independently by two reviewers. Any disagreements were resolved through discussion and re-examination of the original publications.

For the assessment of the RRI measured in the main renal artery, the search was performed using combinations of the terms referring to the renal hilum and main renal vessels together with Doppler-derived resistive index terminology (“main renal artery” OR “renal artery” OR hilar OR hilus OR “renal hilum”) AND (“resistive index”[title and abstract (tiab)] OR “resistance index”[tiab] OR RRI[tiab] OR Pourcelot[tiab]) AND (Doppler OR doppler) NOT (transplant[tiab]) AND (“humans”[MeSH Terms]).

For the evaluation of RRI in segmental and interlobar renal arteries, keywords related to intrarenal Doppler examination were used (intrarenal OR interlobar OR segmental OR sinus) AND (“resistive index”[tiab] OR “resistance index”[tiab] OR RRI[tiab] OR Pourcelot[tiab]) AND (Doppler OR doppler) NOT (transplant) AND (“humans”[MeSH Terms]).

For the analysis of RRI assessed in intraparenchymal and cortical arteries, search terms referring to parenchymal, arcuate, cortical, or interlobular vessels were applied (intraparenchymal OR parenchymal OR arcuate OR cortical OR interlobular) AND (“resistive index”[tiab] OR “resistance index”[tiab] OR RRI[tiab] OR Pourcelot[tiab]) AND (Doppler OR doppler) NOT (transplant) AND (“humans”[MeSH Terms]).

The following PubMed publication types were excluded: address, biography, books and documents, case reports, introductory journal article, lecture, letter, Meta-Analysis and Network Meta-Analysis, news, observational study in veterinary, personal narrative, patient education handout, comment, conference proceedings, consensus statement, corrected and republished article, dataset, practical guideline, preprint, published erratum, duplicate publication, editorial, english abstract only, evidence synthesis, retracted publication, retraction notice, review, scoping review, systematic review, video-audio -media, webcast, expression of concern, festrschrift, guideline, historical article, interview, conference abstracts. Additionally, exclusion criteria included renal transplantation, because of the known influence of altered hemodynamics and graft-specific factors on RRI values, as well as studies performed in non-human subjects. Publications in which the exact site of the RRI measurement was not clearly specified, off-topic, or with different organ-side measurements, arterial stenosis, age < 18 and pregnancy were excluded. Also, studies with a number of participants < 30 were not included, to reduce concerns regarding low statistical power and imprecise estimates of associations in very small samples. Articles describing an RRI measurement at two or three locations—which, based on the search criteria, were initially classified under only one location—were re-included in the analysis of the omitted locations as an “additional records inclusion.”

For this scoping review, cardiovascular factors were defined as parameters or clinical conditions that reflect systemic cardiovascular function, hemodynamics, or vascular alterations. These included, among others, hypertension severity, cardiovascular risk, pulse wave velocity (PWV), blood pressure, cardiac dysfunction, atherosclerosis, endothelial dysfunction, and coronary artery disease. Renal factors were defined as parameters or clinical conditions that reflect kidney function, kidney injury, or structural and vascular alterations within the kidney. These included, among others, renal function, albuminuria, structural and vascular changes within the kidney, intrarenal arteriosclerosis, intrarenal vascular stiffness, kidney biopsy findings, and prediction of acute kidney injury (AKI). These categories were used to organize the variables assessed in the included studies rather than to distinguish mutually exclusive biological mechanisms. An association classified as renal or cardiovascular/hemodynamic should therefore not be interpreted as evidence that RRI exclusively reflects the corresponding compartment. The literature review presented here was conducted in accordance with the PRISMA scoping review guidelines [Supplementary Table S1].

The findings would be synthesized narratively, considering the reported effect estimates, their direction, and whether associations would remain significant after adjustment for potential confounders, where available. Because the included studies use heterogeneous statistical measures and assess different outcomes, no formal classification or standardized comparison of association strength would be performed. Study counts would be used to describe the distribution of the available evidence across measurement sites and association categories, rather than to infer the relative strength of renal and cardiovascular associations.

## 3. Results

**Figure 1:**
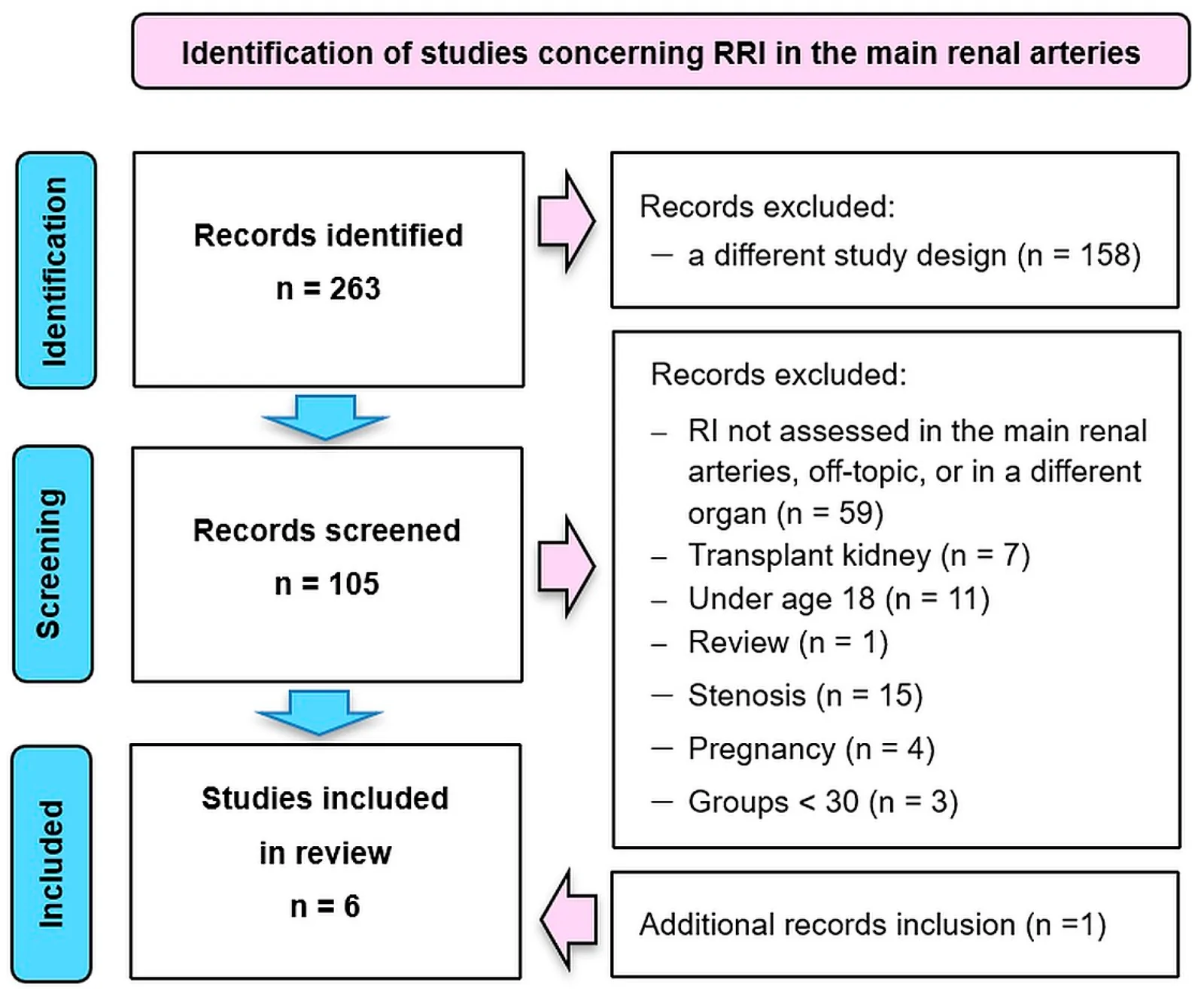
Identification of studies linking RRI in the main renal arteries to cardiovascular and renal markers.

**Figure 2:**
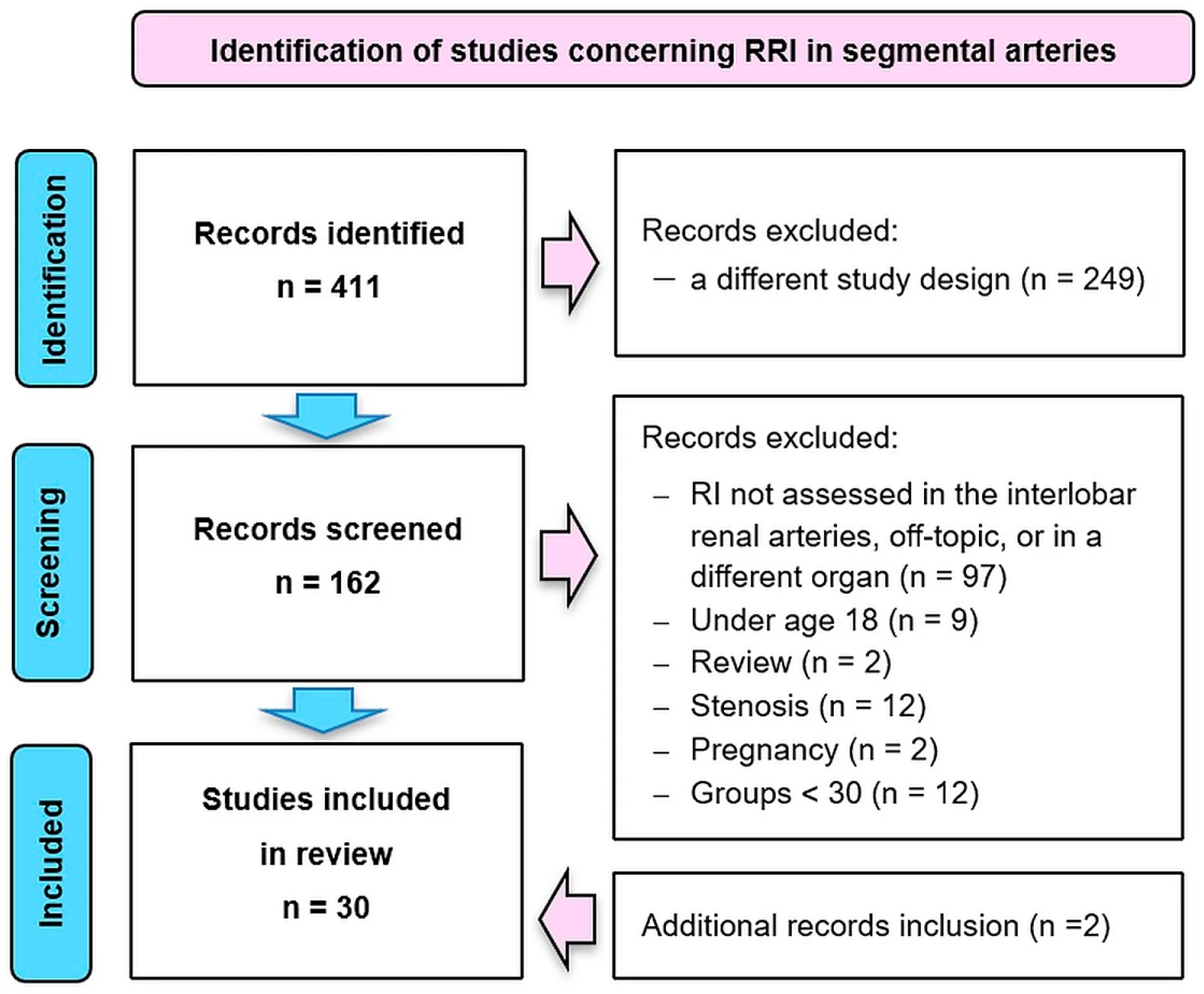
Identification of studies linking RRI in segmental and interlobar arteries to cardiovascular and renal markers.

**Figure 3:**
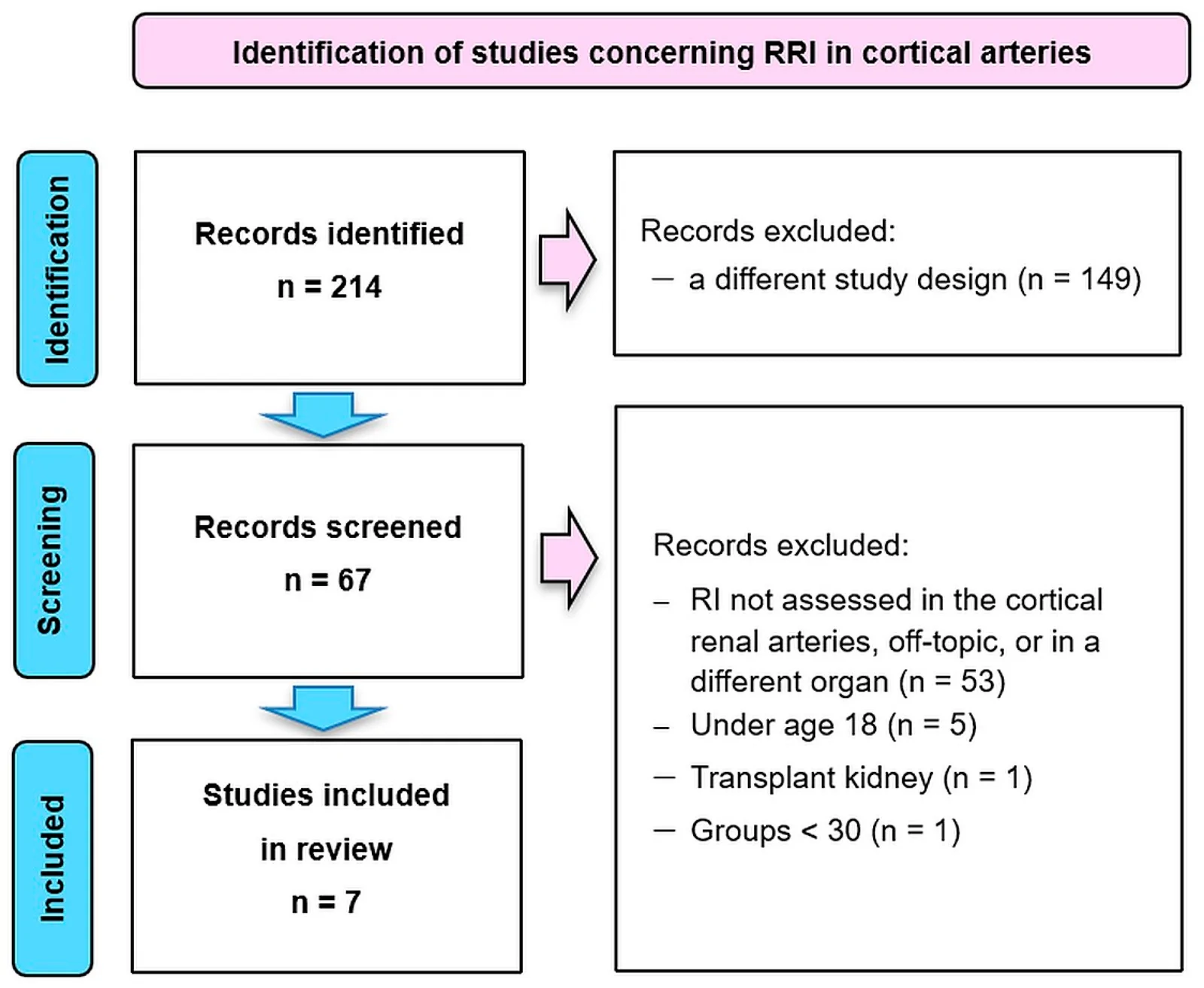
Identification of studies linking RRI in cortical renal arteries to cardiovascular and renal markers.

For articles evaluating the RRI in the main renal arteries, including hilar measurements, a total of 263 publications were identified. For studies assessing the RRI in segmental or interlobar renal arteries, 411 publications were found. In the case of the RRI measured in intraparenchymal and cortical renal vessels, 214 publications met the search criteria. All results are presented in the form of flowcharts (Figure 1, Figure 2 and Figure 3).

## 4. Discussion

### 4.1. Main Renal Artery

After a PubMed search, 6 of 263 publications were included in the final analysis. The majority of studies focused on renal artery stenosis, which was beyond the scope of this analysis, suggesting that measurements at this location are more commonly applied in stenotic pathology than in the assessment of general hemodynamics, cardiovascular or renal function. Among the 6 selected studies, one of them investigated the potential relationship between the RRI and renal structure and function. In this work, Yura et al. examined 78 patients with chronic glomerulonephritis using Doppler sonography [14]. The results showed a correlation between creatinine clearance and the RRI measured at the renal hilus (r = −0.74). In this study, among patients with lower creatinine clearance, the renal resistive index was increased, suggesting that measuring the RRI at the main renal artery may correlate with impaired renal function and reflect hemodynamic changes associated with chronic glomerulonephritis.

Also, Kurihara et al. reported a possible correlation between the RRI and estimated glomerular filtration rate (eGFR, r = −0.627; *p* < 0.001) [15]. The mean RRI was significantly higher (*p* = 0.015) in patients with chronic kidney disease (CKD) than in the healthy group. This supports the conclusion from previous studies that the RRI measured at the main renal artery may be associated with kidney function. However, in CKD, patients exhibit hemodynamic alterations resulting from compensatory mechanisms, such as increased Renin–Angiotensin–Aldosterone system (RAA) activity, which increases blood pressure and may accelerate vascular changes, such as arterial stiffness, that may also contribute to an elevated RRI. On the other hand, the correlation with eGFR, which is a marker of renal function, can be more strongly emphasized there. Conversely, Frascisco et al. showed that resistive index increased in parallel with severity of hypertension, as assessed by mean arterial pressure (*p* < 0.05) [16]. Nevertheless, although the angiotensin-converting enzyme inhibitor (ACE-I) was administered for hypertension, the RRI values were still elevated because of the possible reduced capability of renal blood flow autoregulation, as a result of vascular damage. Therefore, in this case, the RRI values could reflect an association with hemodynamic alterations, such as higher blood pressure, which accelerates arterial stiffening. Moreover, Pearce et al. showed that patients with an RRI ≥ 0.8 had higher systolic blood pressure, lower diastolic pressure, and decreased eGFR ≤ 60 mL/min/1.72 m^2^ [17]. This may suggest that measuring the RRI at the hilar artery of the kidney may be associated with hemodynamic processes, such as hypertension, and with decreased renal function. However, after more complex statistical analysis that accounted for the impact of other factors, such as diabetes, the results showed that end-diastolic frequency shift from Doppler samples obtained from the renal parenchyma could better predict cardiovascular events than the RRI of the hilar arteries. Therefore, the final conclusion suggests that there may be no independent relationship between an RRI ≥ 0.8 at the hilar level and elevated cardiovascular risk, and that parenchymal Doppler measurements likely better reflect these events. On the other hand, Ohta et al. demonstrated that RRI measurements at the main renal artery may be useful for assessing both systemic hemodynamic conditions and renal function [7]. They reported a significant correlation between the RRI in the main renal arteries and pulse wave velocity (PWV), which is a marker of arterial stiffness. This demonstrates the role of the RRI as an indicator of systemic vascular hemodynamic alterations. Also, elevated RRI values in this study were observed in patients with diabetic nephropathy, indicating that the RRI may also reflect structural changes within the kidney associated with chronic disease. Rivolta et al. also assessed the RRI in the main renal arteries [18]. This study is included in Table 1 and discussed in detail in Section 4.4 because it evaluated all three measurement sites within the same patients.

Taken together, these findings may indicate that the RRI measured at the main renal artery reflects both systemic hemodynamic conditions and, to some extent, renal structural alterations (Table 1). However, the number of available studies is limited, likely due to technical difficulties in visualizing the main renal artery during Doppler ultrasonography. As a result, RRI measurements are more commonly performed in intrarenal arteries, where assessment is technically easier, is more often established in clinical practice. Therefore, further studies focusing on RRI measurements in the main renal artery are required to more strictly determine whether this parameter is more closely related to renal function, cardiovascular events, or systemic hemodynamic conditions.

### 4.2. Interlobar and Segmental Arteries

The majority of studies showed a possible association between RRI measurements in the interlobar and segmental arteries and renal structure or function [19,20,21,22,23,24,25,26,27,28,29]. Kaneko et al. examined 132 patients with CKD who underwent renal biopsy, and most had IgA nephropathy [20]. Spearman’s correlation revealed strong positive correlations between the percentage of renal sinus fat (RSF%) and the RRI (r = 0.40; *p* < 0.001) and between the proportion of global glomerular sclerosis (r = 0.48; *p* < 0.001) and interstitial fibrosis with tubular atrophy (IF/TA) (r = 0.35; *p* < 0.001). In this work, RSF volume was assessed using a semi-automated method that involved manual slice-by-slice segmentation of CT axial images. Also, the multivariable linear regression models, after including age, sex, BMI, diabetes, and hypertension, showed an independent association between RSF% and the RRI (β = 0.27; *p* = 0.004). Therefore, renal sinus fat could be associated with renal structural damage in CKD patients, as increased RSF% correlated with a higher RRI in the segmental arteries. However, the study did not examine the relationship between the RRI and hemodynamic markers, such as cardiac output or pulse wave velocity; therefore, it remains unclear whether an increased RRI was associated only with renal structural damage or also with systemic hemodynamic changes. Moreover, Matsumoto et al. showed that the RRI was elevated in patients with diabetic chronic kidney disease (CKD) compared with non-diabetic patients (*p* < 0.0001) [23]. The RRI in diabetic patients was 0.827 ± 0.064, whereas in non-diabetic patients, it was 0.725 ± 0.083. The authors performed multiple regression analysis to test the possible influence of age, total cholesterol, serum triglycerides, albumin, and mean blood pressure, and revealed that RRI values were significantly affected by the presence of diabetes, estimated creatinine clearance, and age (R^2^ = 0.559, *p* < 0.0001). Therefore, this did not indicate a possible association between the RRI and systemic hemodynamic processes, such as blood pressure, but it did show that measuring the RRI at interlobar arteries may correlate with diabetes in patients with CKD, as it may lead to intrarenal arteriosclerosis and interstitial lesions. Furthermore, Viazzi et al. examined 418 patients with untreated primary hypertension and found that those with elevated serum uric acid levels had a higher RRI and higher prevalence of microalbuminuria [21]. It was stated that uric acid was directly related to the RRI (r = 0.24; *p* = 0.007) in women and to albuminuria (r = 0.13; *p* = 0.04) in men. This suggests that an elevated RRI in the interlobar renal arteries may be associated with increased uric acid, a marker of worsening kidney function. However, according to the authors, this relationship was independent of the eGFR. It is hypothesized that mild hyperuricemia, combined with hypertension, might actively cause endothelial dysfunction and arteriolar stiffness, leading to increased albuminuria and structural changes even before glomerular filtration drops. As shown, this increase may correlate with a higher RRI and microalbuminuria; therefore, the RRI may reflect structural kidney damage, although it is likely secondary to vascular alterations. Also, Lubas et al. demonstrated that the RRI measured in segmental arteries correlates with renal cortical blood flow (CBF), which is independently related to eGFR [29]. The association of RRI with eGFR was observed (r = −0.459; *p* = 0.012), suggesting that measuring resistive index in segmental arteries may reflect kidney function. Given that this work also measures the RRI in cortical arteries, it is discussed in greater detail in Section 4.3.

Conversely, some studies have proposed a possible relationship between RRI measurements in the interlobar arteries and systemic hemodynamics [30,31,32,33,34,35,36,37]. In Ponte et al.’s family-based cross-sectional study on 726 patients, univariable analyses showed higher RRI values in hypertensive than in normotensive participants (0.66 ± 0.06 vs. 0.62 ± 0.05; *p* < 0.001) [30]. Also, results from the fully adjusted multivariate model including eGFR, lipids, smoking, hypertension treatment, and diabetes mellitus showed no independent association with the RRI. Nevertheless, the backward stepwise regression revealed positive relationships of the RRI with age, SBP, and BMI, and negative associations with heart rate and DBP. These observations are consistent with the thesis that measuring the RRI in the interlobar arteries can reflect hemodynamic processes, particularly those associated with systolic blood pressure. This suggests that the RRI of interlobar arteries may be a better hemodynamic marker than a renal function marker since no independent association between eGFR and the RRI was observed. However, the formula for calculating eGFR takes into account not only the creatinine concentration, but also age and gender. Therefore, when age is included in the regression model, eGFR may be less strongly associated with the RRI, as part of the relationship is attributed to age. Therefore, it cannot be definitively concluded that there was no relationship with renal function, as both the calculation of this function and the incorrect model construction are subject to significant error. Similar results were observed in a study by MacIsaac et al.in which 167 patients with type 2 diabetes were examined [31]. The mean RRI measured in the interlobar arteries was 0.69 ± 0.01, and 48% of participants had an increased RRI beyond the normal range (>0.7). Also, RRI values were elevated in patients with (any) left ventricular diastolic dysfunction (0.72 ± 0.06). After correcting for multiple clinical and biochemical variables (age, duration of diabetes, BMI, blood pressure, albumin excretion rate, eGFR levels, use of RAA blockers, systolic function), the correlation between the RRI and markers of diastolic dysfunction remained significant (*p* < 0.001). Therefore, these findings may suggest that an elevated RRI in interlobar arteries reflects cardiovascular hemodynamic abnormalities associated with left ventricular diastolic dysfunction, independently of renal function and the presence of CKD. Nevertheless, it remains unclear whether the RRI was significantly associated with kidney function.

Furthermore, in some studies, measuring the RRI in the interlobar arteries showed a possible relationship with both systemic hemodynamic changes and renal structure or function [7,38,39,40,41,42,43,44]. In the multivariable logistic regression model, Toledo et al. showed that older age, female gender, higher systolic blood pressure, coronary artery disease, peripheral vascular disease, and use of β-blockers were associated with higher odds of having an RRI ≥ 0.70 [43]. Moreover, patients with such an RRI had decreased eGFR values. This means that the RRI measured in interlobar arteries may be associated with systemic hemodynamic abnormalities and impaired renal function. Higher RRI values were associated with lower eGFR, indicating reduced renal function, whereas the association with elevated systolic blood pressure may reflect systemic vascular stiffness and hemodynamic disturbances. However, proteinuria, which is also one of the markers reflecting renal injury, was not associated with the RRI. At the same time, diabetes mellitus, hyperlipidemia, coronary artery disease, and congestive heart failure were more prevalent in patients with an RRI ≥ 0.70. Therefore, this may indicate that the correlation of the RRI with diverse systemic alterations is stronger than its association with renal function alone. Furthermore, Boeri et al. examined 36 patients with non-insulin-dependent diabetes mellitus (NIDDM) and found that the RRI in intrarenal arteries was higher in diabetic patients (median 0.72, range 0.54–0.79 than in the control group (median 0.62, range 0.57–0.66) [41]. The correlation between the RRI and age (*p* < 0.004), body mass index (r = 0.598; *p* < 0.001), mean blood pressure (r = 0.561; *p* < 0.001), total cholesterol (r = 0.578; *p* < 0.01), and creatinine clearance (r = 0.491; *p* < 0.01) was observed. These findings suggest a relationship between the RRI and renal function, as well as with hemodynamic and other systemic disturbances. Measuring the RRI in the intrarenal arteries showed a possible correlation with cardiovascular risk factors [45,46]. In the study conducted by Geraci et al., patients with an RRI ≥ 0.70 had significantly lower eGFR values (60.36 vs. 77.05 mL/min/1.73 m^2^; *p* < 0.001), as well as higher RRI values were observed in participants with greater 10-year risk of cardiovascular (CV) events (assessed by Framingham Risk Score (RRI > 0.67) or Atherosclerotic Cardiovascular Disease (ASCVD) Risk Score (RRI > 0.65) in the overall study population) (*p* < 0.001) [45]. The multivariate analysis, with Framingham Risk Score as the outcome variable, revealed that an RRI was independently associated with this CV risk score. Therefore, this suggests that the RRI in the intrarenal arteries may be used to predict the risk of CV events, with higher RRI values associated with greater risk, reflecting its relationship with cardiovascular alterations. Moreover, in the study conducted by Geraci et al., an increase in the RRI was observed across groups with progressively more severe carotid atherosclerosis (0.61 ± 0.07, 0.65 ± 0.06, 0.68 ± 0.06; *p* < 0.001) [46]. A multivariable analysis of covariance, adjusting for potential confounding factors such as age, serum glucose, smoking habit, serum uric acid, BMI, HDLc, triglycerides, albuminuria, eGFR, and 24 h pulse pressure, performed in 3 groups with different severity of carotid pathology revealed stepwise increased RRI values of 0.63 ± 0.06, 0.65 ± 0.06, and 0.66 ± 0.06. These suggest that the RRI in the interlobar arteries may serve as a marker of systemic vascular changes, as it correlates with carotid atherosclerosis. Rivolta et al. also assessed an interlobar RRI [18]. This study is included in Table 2, with a detailed discussion of its findings across all three measurement sites provided in Section 4.4.

Across these papers, most focused on the possible correlation between the RRI measured in the interlobar arteries and renal function (Table 2). However, other studies also suggested the possible relationship between the RRI and systemic hemodynamic abnormalities, and some of them with cardiovascular alterations. Nevertheless, it remains unclear whether RRI measurements obtained at different locations, such as the main renal arteries or the kidney’s parenchymal arteries, would differ. Since these comparisons were not directly investigated, it is possible that they would have similar values and correlate with hemodynamic processes or renal function in these patients. However, we cannot determine this, as it was not examined and requires further evaluation. Also, not all studies performed multivariable analyses, and in some, it is not possible to determine whether the observed relationships were independent.

### 4.3. Interlobular and Arcuate Arteries

In the 7 studies that met the final criteria for the review, the association between cortical arterial RRI and both renal structure or function and systemic hemodynamic alterations was investigated, with a noticeable inclination toward the former. Hu et al. evaluated the application of acoustic radiation force impulse and conventional ultrasonography in the quantitative assessment of IgA nephropathy, and demonstrated a significant correlation of shear wave velocity (SWV), renal length, renal parenchymal thickness and RRI measured in interlobular arteries with Katafuchi grading, mesangial hypercellularity and IF/TA (r = –0.504 to –0.407, *p* < 0.01), yet no association with endocapillary hypercellularity or segmental glomerulosclerosis [47]. Although the area under the curve for the combined assessed parameters was higher than for SWV or conventional ultrasound indices (renal length, renal parenchymal thickness, RRI) alone, the study confirmed a relationship between an interlobular RRI and IgA-associated intrarenal pathology. Furthermore, Lubas et al. demonstrated that dynamic RI (dRI) measured in the renal cortex, along with RRI assessed in segmental arteries, correlates with renal cortical blood flow (CBF), which, in turn, is independently related to eGFR [29]. The association of dRI and RRI with eGFR was also observed, and dRI appeared to correlate slightly more strongly (r = −0.534; *p* = 0.002 vs. r = −0.459; *p* = 0.012), suggesting that measuring resistive index in cortical arteries rather than in segmental arteries better reflects kidney function. However, the difference was not significant, and further research would be needed to confirm this assumption. It should be noted that in this study neither the RRI nor dRI were independently associated with eGFR, suggesting that CBF and end-diastolic velocity (EDV) are the only independent markers. In terms of CBF, the RRI seemed to best illustrate it among conventional Doppler parameters, yet among all the considered ultrasound parameters, dRCP (dynamic renal cortex perfusion) emerged as the independent marker. The predictive value of the RRI was investigated in a study by Wybraniec et al., who found an association between a pre-procedural cortical RRI and contrast-induced acute kidney injury (CI-AKI), defined as a ≥50% relative or ≥0.3 mg/dL absolute increase in serum creatinine 48 h after contrast agent administration [48]. Patients in whom CI-AKI was later diagnosed had significantly higher RRI values (0.69 vs. 0.62; *p* = 0.005). This shows that the RRI, as a potential marker of subclinical renal vascular pathologies, may be seen as a risk factor for CI-AKI in patients with originally preserved renal function, and raises the question of the usefulness of this parameter in assessing the risk of acute kidney injury, in general, as a possible area of further investigation.

Another study carried out by Wybraniec et al. seems to, conversely, demonstrate the relation between the RRI in arcuate/interlobular arteries and systemic hemodynamic changes, as it shows association of this index to prognosis in patients with coronary artery disease (CAD) [49]. The investigators tested 111 patients with stable or unstable CAD, aiming to assess the usefulness of intrarenal Doppler flow indices, as well as the RRI and RPI (renal pulsatility index), in predicting major adverse cardiac and cerebrovascular events (MACCE: cardiovascular death, myocardial infarction, myocardial revascularization or stroke). During a 24-month follow-up, MACCE occurred in 14 patients (12,6%). Pre-procedural and post-procedural RRI values in those patients were significantly higher (0.68 ± 0.06 vs. 0.62 ± 0.06, *p* < 0.001; and 0.72 ± 0.06 vs. 0.66 ± 0.06, *p* = 0.01, respectively), while the RPI values were comparable between groups with and without MACCE. These results show that an arcuate/interlobular arterial RRI is a valuable marker of systemic cardiovascular alterations, rather than solely of intrarenal microcirculation, as demonstrated in the previously mentioned studies. A study by Fukumoto et al. provides a notable contribution to the topic, although their results do not directly point towards the RRI’s connection to local or systemic hemodynamic changes [50]. This study evaluated the association between the RRI of the arcuate arteries and the angiotensin-converting enzyme (ACE) gene polymorphism in 32 patients with insulin-dependent diabetes mellitus (IDDM) without symptoms of nephropathy. The RRI was significantly higher in patients with DD (deletion/deletion) genotype (0.71 ± 0.05), which is normally responsible for higher ACE activity, than in patients with ID (insertion/deletion) or II (insertion/insertion) genotypes (0.64 ± 0.04 and 0.66 ± 0.05, respectively; II vs. DD, *p* < 0.02), associated with lower activity of the enzyme. Surprisingly, multivariable regression analysis showed that ACE genotype was the only one correlated with an arcuate arterial RRI, among various types of parameters (sex, age, IDDM duration, body mass index, HbA1c, plasma glucose levels, serum levels of total cholesterol and creatinine, urinary albumin excretion index, mean blood pressure). In addition, the researchers found no correlation between the ACE genotype and RRI of interlobar arteries. These results might indicate that, as Fukumoto et al. suggest, renal cortical resistive index is arguably one of the best markers of intrarenal hemodynamic changes and a notable predictor of diabetic nephropathy. Nonetheless, as the study comprised only patients with IDDM, it cannot be clearly stated whether the RRI is associated solely with diabetic nephropathy, as investigated here, or with other types of nephropathy as well, particularly given that the influence of the ACE gene polymorphism on the development of diabetic nephropathy is not evident. It is possible that the RRI’s relation to ACE genotype results from the impact that ACE has on intrarenal microcirculation independently of diabetes mellitus, or even systemic hypertensive effects of this enzyme and its product, angiotensin II. Such an interpretation would present the RRI of arcuate arteries as a valuable marker of early systemic hemodynamic changes, arguably more sensitive than an interlobar RRI, which showed no correlation with ACE genotype in Fukumoto’s research. It has to be borne in mind, however, that the investigated group in the discussed study is specific and small, and broader research is needed to confirm the connections found and clarify the conclusions. The relation between the cortical RRI and hypertension has been, however, investigated in a study by Caruso et al., who tested the effects of 12-month treatment with losartan (50 mg/d) and chlorthalidone (25 mg/d) in 92 hypertensive patients, all of whom had an elevated RRI (>0.68) but normal renal function [51]. Of 48 patients treated with losartan, 47 (97.5%) showed normalization of the RRI, whereas only 14 of 44 (25.8%) treated with chlorthalidone showed a similar effect. No significant difference in blood pressure control was observed between the groups. A total of 30 patients, in whom the RRI remained elevated after chlorthalidone therapy, underwent a 2-week washout and then were treated with losartan for another 12 months. 28 of them (95%) had their RRI normalized after this period, despite no difference in BP control. These results, as the researchers state, point to nephroprotective effects of losartan, in addition to its BP-lowering effects. At the same time, although not directly, the connection between a cortical RRI and angiotensin was again shown, potentially contributing to the conclusions from the previously discussed study. Caruso et al., while focusing on the effects of different antihypertensive drugs, demonstrated that the RRI is not directly dependent on blood pressure, suggesting that the resistive index of renal cortical arteries is not the best marker of systemic hemodynamics. On the other hand, no relation between the RRI and renal structure or function was observed in this study, and the conclusion about the nephroprotective effect of losartan is based solely on RRI values, which the authors had previously classified as parameters reflecting intrarenal alterations. Therefore, the question of whether the RRI measured in cortical arteries better correlates with local pathologies than with systemic changes remains open. An additional study by Rivolta et al. reported associations involving a cortical RRI but also assessed an RRI in the main renal and interlobar arteries within the same patients [18]. Given its relevance to comparisons across measurement sites, this study is discussed separately in greater detail below.

Table 3 summarizes studies included in this review that examined the relationship between the RRI measured in renal parenchymal arteries.

### 4.4. RRI Assessed in Each Location of Renal Arterial Tree

An interesting contribution to this area and valuable data concerning the main purpose of this review are provided by Rivolta et al. in a study comparing color Doppler ultrasonographic parameters in the kidneys of healthy volunteers and patients with systemic sclerosis (SSc), all without clinical signs of renal involvement [18]. What is important is that the RRI was measured in the main, interlobar, and cortical arteries. In all localizations, the RRI values were higher in the SSc group and correlated with disease duration. The difference was significant and similar in all the examined sites (70.8 +/− 5.7 [68.5–73.1] vs. 57.6 +/− 4.2 [55.9–59.3], *p* < 0.0001 in main renal arteries, 69.9 +/− 5.8 [67.5–72.3] vs. 55.3 +/− 5.0 [53.2–57.4], *p* < 0.0001 in interlobar arteries, 65.0 +/− 5.9 [62.6–67.4] 51.8 +/− 5.1 [49.7–53.9], *p* < 0.001), yet the correlation with the duration of the disease in the patients with SSc resulted to be the strongest in cortical arteries (main arteries r = 0.56, *p* < 0.04; interlobar arteries r = 0.63, *p* < 0.02; cortical arteries r = 0.75, *p* < 0.002). These results indicate that there may be little or no difference between RRI measurement sites within a specific patient group, but, on the other hand, they may significantly differentiate healthy patients from those with systemic renal disease. There is, however, a difference in how resistive indices examined in different types of vessels reflect advancing alterations in the intrarenal microcirculation, as the findings of Rivolta et al. correspond with the histologic picture of kidneys in the course of SSc. It is worth underlining that no association between the RRI of any vessel and creatinine clearance was found in this study, suggesting the applicability of this parameter, especially when measured in the cortex, in signaling subclinical intrarenal changes and, possibly, predicting clinical symptoms of renal involvement.

### 4.5. The Influence of Antihypertensive Treatment

In many studies, patients enrolled in the analysis are already receiving pharmacological treatment. This therapy most commonly includes antihypertensive agents, such as β-blockers and ACE-I, which may also have nephroprotective effects. Lubas et al. demonstrated a possible association between the RRI measured in the parenchymal arteries and treatment with RAA blockers and β-blockers in patients with chronic kidney disease and hypertension [52]. The multivariable regression analyses adjusted for age showed that diuretics were associated with a decrease (r = −0.473) and RAA blockers (r = 0.390) with improved proximal and whole renal cortex perfusion (R^2^ = 0.28; *p* < 0.001). This may suggest that treatment with RAA blockers, which lowers blood pressure in patients with hypertension, may be associated with improved perfusion of parenchymal arteries, due to vasodilation of efferent arterioles in the renal glomerulus. However, in multivariable regression, in which a cortical RRI was significantly associated with ACE-I and β-blockers (R^2^ = 0.18, *p* < 0.01), the direction of these effects depends strongly on the drug class. While ACE-I treatment may be associated with a decreased RRI and improved cortical perfusion due to vasodilation, the use of B-blockers may be associated with an increased RRI and reduced distal cortical perfusion (R^2^ = 0.21, *p* < 0.01). The RRI is negatively associated with heart rate, and β-blockers lower it by binding to the β1 and β2 receptors, exerting negative chronotropic and inotropic effects on the heart [52]. This mechanism may explain the possible effect of this class of drugs on increasing the RRI. Overall, these findings may suggest a relationship between the RRI measured in parenchymal arteries and the specific pharmacological mechanisms of antihypertensive drugs. Moreover, a study by Delić-Brkljačić et al. found that treating patients with valsartan, an antihypertensive drug, significantly lowers RRI values in interlobar arteries compared with baseline (*p* < 0.001) [34]. Similar effects were observed in participants treated with a different antihypertensive drug, nifedipine, where the RRI decreased significantly after therapy (*p* < 0.001). However, patients treated with valsartan had lower RRI values (0.615 ± 0.036) after six months than those treated with nifedipine (0.642 ± 0.030) (*p* < 0.01). Both types of medicines reduced systolic and diastolic blood pressure equally in patients with essential hypertension, but valsartan lowered the RRI in the renal interlobar arteries to a greater extent than nifedipine did. This suggests that the RRI may reflect hemodynamic changes, such as blood pressure, but the results differ between the two treatments, possibly due to distinct mechanisms of these drugs. Valsartan may have a stronger impact on arteries and a lower RRI, as it acts by blocking the angiotensin II receptor, preventing arterial constriction in the kidney and lowering blood pressure. However, nifedipine is a drug from a different class that blocks calcium channels, resulting in vasodilation; as observed, these two mechanisms may affect the RRI differently. Furthermore, Meco et al. treated 30 patients undergoing cardiac surgery with fenoldopam mesylate and examined its effects on hemodynamic and renal parameters [53]. The results revealed significant increases in PSV (53.2%) and EDV (78.2%) measured at the main renal artery. However, the RRI measured at this level of the renal tree decreased significantly. At baseline, the RRI was 0.7 ± 0.1, and after fenoldopam infusion, it was 0.6 ± 0.1 (*p* < 0.01). This may suggest a possible association between this drug and a reduction in the RRI of the main renal arteries. This may be due to fenoldopam’s mechanism as a selective agonist at dopamine D1 receptors. As this drug acts by activating these receptors, it causes arterial vasodilation and, in effect, lowers the RRI. However, variables used to examine hemodynamic changes after drug infusion, such as heart rate, arterial pressure, and preload indices, did not change significantly. Therefore, it may suggest that fenoldopam, despite its influence on the RRI in the main renal artery, does not correlate with changes in general hemodynamic parameters (which may also influence the RRI). Thus, it is possible that this drug may affect RRI values independently through its specific mechanism of action. In relation to the main question of this review, the available studies do not establish whether drug-induced changes in the RRI differ by measurement site. Although treatment-related changes were reported at different levels of the renal arterial tree, the studies used different medications and included different patient populations. Therefore, these findings cannot determine whether any measurement site is more sensitive to treatment or whether the observed differences primarily reflect drug-specific mechanisms. Studies assessing the same treatment and measuring the RRI at multiple sites within the same patients are needed to distinguish these effects.

### 4.6. Limitations

Nevertheless, our review has some limitations. The main limitation of this review is that comparisons across RRI measurement sites are based predominantly on separate studies rather than measurements obtained at different vascular levels within the same patients. The included studies differed substantially in patient populations, clinical conditions, assessed variables, outcomes, and statistical approaches. Some studies were specifically designed to investigate the association between the RRI and either renal or cardiovascular/hemodynamic factors and did not simultaneously evaluate variables representing the other category. Therefore, the absence of a reported association with renal or cardiovascular factors should not necessarily be interpreted as evidence of no such association, as the corresponding parameters may not have been assessed. Consequently, differences in the reported associations may reflect these study characteristics as well as the measurement site, and their respective contributions cannot be separated. This review therefore describes patterns of association reported in the literature but cannot establish that these patterns are directly attributable to the location of the RRI measurement. Also, in Doppler ultrasonography of renal arteries, not only the RRI but also hemodynamic parameters, such as peak systolic velocity (PSV), end-diastolic velocity (EDV), pulsatility index (PI), acceleration time (AT), and acceleration index (AI), could be assessed, providing complementary information about renal blood flow, vascular resistance, and renal hemodynamics. Therefore, in further studies, these markers should be considered. Regarding the study selection process for the review, we searched only in the PubMed database. Although it is the largest, some valuable studies available in other databases that are not accessible via PubMed might be missing from our work. This limitation is particularly relevant to the main renal artery and cortical vessel categories, which included only six and seven studies, respectively. Relevant studies included in other databases may have been missed, potentially affecting the completeness of the evidence and the patterns of association described for these measurement sites. Moreover, we excluded studies performed on small groups (<30 participants). Although this scoping review aimed to map the available evidence, it also sought to compare reported associations between the RRI and renal or cardiovascular parameters across measurement sites. Very small samples may yield imprecise estimates and have limited power to detect associations, making both positive and negative findings difficult to interpret comparatively. The minimum sample size criterion was therefore retained to reduce these concerns. However, 30 participants should not be regarded as a universal threshold for adequate statistical power or methodological quality. We acknowledge that smaller studies may provide useful information and that their exclusion may have reduced the completeness of the evidence, particularly for measurement sites with limited literature. Despite this criterion, the studies included in the review ultimately comprised very diverse groups of patients, ranging from 30 to 1962 records per study. These differences affect the credibility of the comparison between the studies and suggest that, in the future, similar research, including a larger number of patients, should be conducted to confirm our conclusions. Additionally, the number of studies assigned to each type of vessel differs, as the discussions concerning the main renal artery and renal cortical arteries include only 6 and 7 studies, while the number of works qualified for segmental arteries was four times higher. The small number of studies assessing the cortical RRI may be explained by technical challenges of obtaining adequate Doppler waveforms from small parenchymal vessels. However, our search strategy and eligibility criteria may also have contributed to the limited number of studies included in this category. Consequently, the available evidence is insufficient to draw firm conclusions about the diagnostic and prognostic value of the cortical RRI. This uncertainty should not be interpreted as evidence of limited clinical utility. Rather, it highlights the need for further studies using standardized measurement protocols and directly comparing reproducibility across measurement sites. Also, the included studies come from different time periods, from studies conducted in the 1990s to those published in recent years. Consequently, differences in ultrasound equipment, Doppler technology and measurement methodology may have contributed to heterogeneity across studies and may limit their direct comparability. Furthermore, some of the studies initially considered for inclusion did not clearly specify the RRI measurement site. Due to the lack of this information, these studies could not be reliably assigned to any of the predefined measurement site categories and therefore could not be included in the review, despite the possibility that some of them contained potentially relevant data that might have contributed additional information to our findings. Finally, in many of the studies considered, multivariable analyses were not conducted. Moreover, some studies did not simultaneously evaluate the associations of the RRI with systemic hemodynamics and renal injury markers. Therefore, we did not perform a meta-analysis, and adequate comparisons of RRI relationships were very difficult. After considering these limitations, a prospective study should be conducted to determine whether the RRI is more strongly associated with hemodynamic parameters or renal function, or whether the measurement site has a significant impact.

### 4.7. Summary

**Figure 4:**
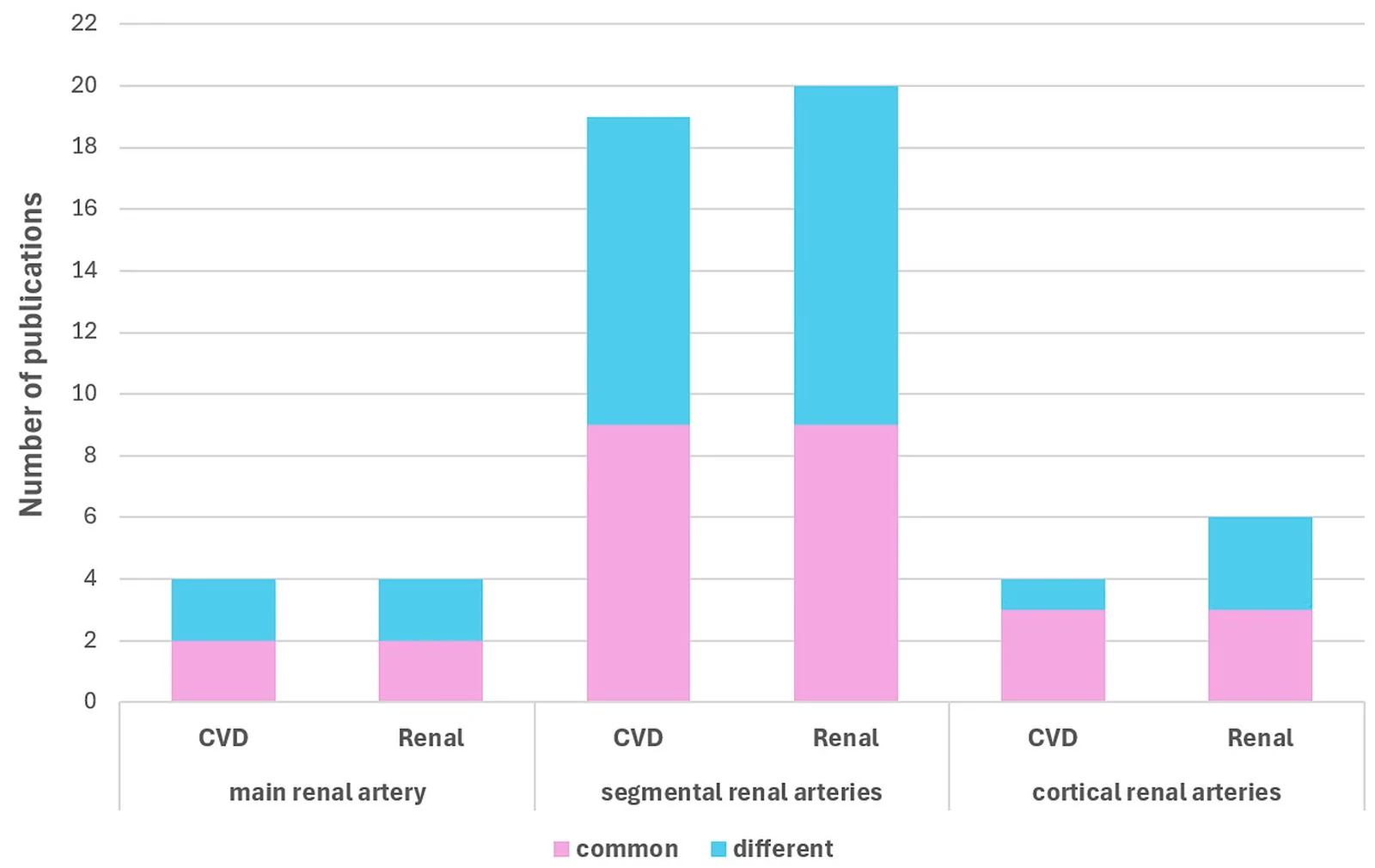
Graphical presentation of the number of publications referring to the considered RRI measurement site, categorized by reported association with cardiovascular diseases (CVD) or renal structural or functional alterations (different), or by association with both (common).

The association between the RRI measured in the main renal arteries and cardiovascular parameters was highlighted in 2 studies; the correlation with renal function was observed in 2 studies. Another 2 papers described the association between RRI and kidney and hemodynamic processes simultaneously (Figure 4).

Multivariate analyses were conducted only in two studies that showed a possible correlation between the RRI and renal structure and function, but not in the papers reflecting hemodynamic associations. This may limit direct comparison of the strength of these relationships. Therefore, it is not possible to determine whether measuring the RRI in the main arteries reflects renal function and structure, or hemodynamic processes. Among the studies focusing on the RRI measured in segmental or interlobar arteries, 9 were associated with both renal and cardiovascular factors, while 11 focused solely on the former, and 10 on the latter. These counts describe the distribution of reported associations but do not establish whether an interlobar/segmental RRI is more strongly associated with systemic or renal alterations. However, some studies suggest that the association with cardiovascular changes might be stronger, as the RRI shows no independent correlation with eGFR (Ponte et al.) or a relationship with markers of renal function, such as proteinuria (Toledo et al.). In spite of that, several studies still demonstrate the RRI of segmental and interlobar arteries as a valuable marker of intrarenal pathologies, and a definitive answer on whether it better reflects systemic or local changes remains unclear. Among articles focused on measuring the RRI in parenchymal arteries, 3 describe possible associations with both renal function and cardiovascular factors, only 1 reports a correlation with hemodynamics, and 3 show a connection between RRI and the kidney. This may suggest that the RRI in parenchymal arteries is more strongly associated with parameters such as IF/TA (interstitial fibrosis/tubular atrophy) and eGFR, which reflect renal function, than with general hemodynamic properties, such as SBP, DBP, and PWV. These patterns should be interpreted cautiously, as they arise mainly from comparisons between heterogeneous studies and may reflect differences in the populations and variables investigated rather than differences attributable to the RRI measurement site. Their relative strength cannot be established from the number of studies reporting each type of association. Moreover, our review revealed a possible association between the RRI and various treatments, particularly antihypertensive medications such as beta-blockers, RAAS blockers (e.g., valsartan), diuretics, and fenoldopam mesylate. All the analyzed papers showed that at each site of the renal artery tree (main renal arteries, segmental arteries, and parenchymal arteries), RRI values decreased after starting antihypertensive treatment, except for beta-blockers, which were reported to increase the RRI in one study. However, it is difficult to determine which RRI measurement site is most sensitive to treatment, because different medications were used across studies, their mechanisms differed, and some had a stronger effect on lowering the RRI than others. Furthermore, no two studies applied the same type of treatment and measured the RRI at more than one location, so the observed changes in the RRI may not be specific to a single assessed site.

## 5. Conclusions

In conclusion, the available literature does not allow us to provide a clear answer to the question of how RRI measurements differ across sites in signaling systemic or local alterations. Some reported patterns are consistent with the hypothesis that the associations of the RRI with renal and systemic hemodynamic parameters may vary by measurement site, suggesting that the RRI measured closer to the kidney may be more closely associated with kidney-specific pathologies, whereas the RRI measured in more proximal arteries may better reflect hemodynamic changes. However, these patterns arise mainly from indirect comparisons between heterogeneous studies and should be interpreted cautiously. Current evidence does not establish whether RRI measurements obtained at different sites can be used interchangeably. To confirm or refute this premise, a study that directly compares the RRI across all three locations and assesses its association with systemic and renal changes is needed.

## Figures and Tables

**Table 1:** Studies examining the relationship between RRI measured in main renal arteries and cardiovascular and renal factors.

Cardiovascular Factors				Renal Factors			
Study	n	Population	RRI Association	Study	n	Population	RRI Association
Frascisco et al. [16]	30	HT	Hypertension severity	Yura et al. [14]	78	Chronic glomerulonephritis	Impaired RF
Pearce et al. [17]	726	Elderly Americans	SBP	Kurihara et al. [15]	36	CKD and non-CKD	RF
Ohta et al. [7]	245	Atherosclerosis, DKD	PWV	Ohta et al. [7]	245	Atherosclerosis, DKD	Structural changes within the kidney
Rivolta et al. [18]	50	Healthy/SSc	SSc, SSc duration	Rivolta et al. [18]	50	Healthy/SSc	SSc, SSc duration

CKD—chronic kidney disease; DKD—diabetic kidney disease; HT—hypertension; PWV—pulse wave velocity; RF—renal function; SBP—systolic blood pressure; SSc—systemic sclerosis.

**Table 2:** Studies examining the relationship between RRI measured in segmental arteries and cardiovascular and renal factors.

Cardiovascular Factors				Renal Factors			
Study	n	Population	RRI Association	Study	n	Population	RRI Association
Ponte et al. [30]	726	Families of European ancestry	SBP	Mulier et al. [19]	99	Critically ill patients	AKI prediction
MacIsaac et al. [31]	167	T2DM	Cardiac diastolic dysfunction	Kaneko et al. [20]	132	CKD	Kidney biopsy findings (GS, IF/TA)
Jaques et al. [32]	1006	Adult population	Arterial stiffness, atherosclerosis	Viazzi et al. [21]	418	HT	uric acid, MA
Geraci et al. [33]	264	HT	PWV	Watanabe et al. [22]	30	HT	MA
Delić-Brkljačić et al. [34]	134	HT	HT	Matsumoto et al. [23]	90	Nondialyzed CKD	intrarenal arteriosclerosis, IF
Corradi et al. [35]	61	Postoperative Cardiac Surgery Patients	tissue perfusion and hypoxia	Nosadini et al. [28]	157	T2DM with MA	RF
Okura et al. [36]	51	HT	PP, IMT	Marzano et al. [24]	81	Diabetes mellitus	RF
Geraci et al. [37]	476	Elderly HT without overt CVD	IMT, PWV	Yazici et al. [25]	873	Diagnostic iodine contrast administration	Prediction of CI-AKI
Geraci et al. [46]	263	HT with and without impaired RF	Atherosclerosis	Tanimura et al. [26]	175	HT	Hemoglobin/EPO production
Geraci et al. [45]	742	HT without CVD	A marker of overall CV risk	Muraira-Cárdenas et al. [27]	63	T2DM	MA
				Lubas et al. [29]	30	HT	RF, RCP
Jaques et al. [38]	1026	General adult population	SBP	Jaques et al. [38]	1026	General adult population	RF
Derchi et al. [39]	291	HT	SBP, DBP, PP, total cholesterol, IMT	Derchi et al. [39]	291	HT	RF, albuminuria
Kawai et al. [40]	194	Elderly patients	DBP, PP	Kawai et al. [40]	194	Elderly	RF, albuminuria
Ohta et al. [7]	245	Atherosclerosis and DKD	Atherosclerosis	Ohta et al. [7]	245	Atherosclerosis, DKD	DKD, IF/TA
Casadei et al. [44]	150	T2DM	Generalized vascular involvement	Casadei et al. [44]	150	T2DM	Vascular changes in the kidney
Boeri et al. [41]	36	T2DM	MAP, total and LDL cholesterol	Boeri et al. [41]	36	T2DM	RF, intrarenal arteriosclerosis
Gigante et al. [42]	45	SSc	Progression of systemic microvascular damage, endothelial dysfunction	Gigante et al. [42]	45	SSc	Intrarenal arterial stiffness
Toledo et al. [43]	1962	CKD	DBP, SBP, coronary artery disease	Toledo et al. [43]	1962	CKD	RF
Rivolta et al. [18]	50	Healthy/SSc	SSc, SSc duration	Rivolta et al. [18]	50	Healthy/SSc	SSc, SSc duration

CKD—Chronic Kidney Disease; CVD—cardiovascular disease; DBP—diastolic blood pressure; DKD—diabetic kidney disease; EPO—erythropoietin; GS—glomerular sclerosis; HT—hypertension; IF/TA—interstitial fibrosis and tubular atrophy; IMT—Carotid intima-media thickness; MA—microalbuminuria; MAP—mean arterial blood pressure; PP—pulse pressure; PWV—pulse wave velocity; RF—renal function; SBP—systolic blood pressure; T2DM—type 2 diabetes mellitus; SSc—systemic sclerosis; RCP—renal cortical perfusion.

**Table 3:** Studies examining the relationship between RRI measured in parenchymal arteries and cardiovascular and renal factors.

Cardiovascular Factors				Renal Factors			
Study	n	Population	RRI Association	Study	n	Population	RRI Association
Fukumoto et al. [50]	32	IDDM	ACE genotype	Fukumoto et al. [50]	32	IDDM	ACE genotype
Caruso et al. [51]	92	HT	losartan vs. chlorthalidone	Caruso et al. [51]	92	HT	losartan vs. chlortalidone
Rivolta et al. [18]	50	Healthy/SSc	SSc, SSc duration	Rivolta et al. [18]	50	Healthy/SSc	SSc, SSc duration
Wybraniec et al. [49]	111	CAD	MACCE	Lubas et al. [29]	30	HT	RF, RCP
				Wybraniec et al. [48]	95	CA-CAD	CI-AKI
				Hu et al. [47]	146	IgAN	Structural alterations in IgAN

ACE—angiotensin-converting enzyme; CA-CAD—contrast agent coronary artery disease diagnosis; CAD—patients with coronary artery disease; CI-AKI—contrast-induced acute kidney injury; HT—hypertension; IDDM—insulin-dependent diabetes mellitus; IgAN—IgA nephropathy; MACCE—major adverse cardiac and cerebrovascular events; RCP—renal cortical perfusion; RF—renal function; SSc—systemic sclerosis.

## Data Availability

No data were created in this study. Data sharing is not applicable to this article.

## Supplementary Materials

The following supporting information can be downloaded at: https://www.mdpi.com/article/10.3390/jcm15218466/s1, Table S1. PRISMA scoping review checklist.
